# Crystal structure of (4*R*,5*S*)-4-methyl-3-methyl­sulfinyl-5-phenyl-1,3-oxazolidin-2-one

**DOI:** 10.1107/S1600536814024702

**Published:** 2014-11-15

**Authors:** Gustavo Pozza Silveira, Vinicius Flores da Silva, Allen G. Oliver

**Affiliations:** a Universidade Federal do Rio Grande do Sul, Instituto de Química Depto. Química Orgânica, Av. Bento Gonçalves, 9500 Agronomia, CEP 91.501-970, Porto Alegre/RS, Brazil; b University of Notre Dame, Department of Chemistry and Biochemistry, 235 Nieuwland Science Hall, Notre Dame, IN 46556-5670, USA

**Keywords:** crystal structure, oxazolidinone, asymmetric indole

## Abstract

The absolute structure of the chiral asymmetric indole precursor title compound, C_11_H_13_NO_3_S, was confirmed by refinement of the Flack and Hooft parameters and is that expected based on the starting materials for the synthesis. The phenyl group subtends a dihedral angle of 56.40 (5)° with the mean plane of the oxazolidinone ring, which adopts an envelope conformation, with the C atom bearing the methyl group as the flap. In the crystal, no significant directional inter­actions beyond van der Waals contacts are observed.

## Related literature   

For general background to the preparation of naturally occurring alkaloids, see: Marino *et al.* (1992 ▶). For further synthetic details, see: Silveira & Marino, 2013 ▶. For related structures, see: Evans *et al.* (1992 ▶); Silveira *et al.* (2013 ▶); Silveira *et al.* (2012 ▶); Clara-Sosa *et al.* (2004 ▶); Romanenko *et al.* (2003 ▶). A statistical analysis (Hooft *et al.*, 2008 ▶) was used to corroborate that the correct enanti­omorph of the space group and hence handedness of the mol­ecule had been determined.
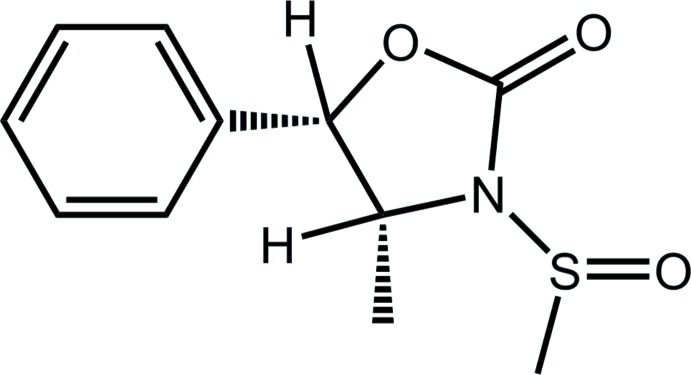


## Experimental   

### Crystal data  

C_11_H_13_NO_3_S
*M* *_r_* = 239.28
Orthorhombic, 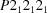

*a* = 6.1605 (4) Å
*b* = 11.8490 (8) Å
*c* = 15.3861 (11) Å
*V* = 1123.12 (13) Å^3^
*Z* = 4
Mo *K*α radiation
μ = 0.28 mm^−1^
*T* = 100 K
0.22 × 0.09 × 0.06 mm

### Data collection  

Bruker X8 APEXII CCD diffractometer
Absorption correction: multi-scan (*SADABS*; Sheldrick, 2007 ▶) *T*_min_ = 0.707, *T*_max_ = 0.746
30938 measured reflections
3761 independent reflections
3507 reflections with *I* > 2σ(*I*)
*R*_int_ = 0.032

### Refinement  

*R*[*F* ^2^ > 2σ(*F* ^2^)] = 0.027
*wR*(*F* ^2^) = 0.070
*S* = 1.03
3761 reflections
147 parameters
H-atom parameters constrained
Δρ_max_ = 0.31 e Å^−3^
Δρ_min_ = −0.18 e Å^−3^
Absolute structure: Flack *x* determined using 1431 quotients [(*I* ^+^)−(*I* ^−^)]/[(*I* ^+^)+(*I* ^−^)] (Parsons *et al.*, 2013 ▶)
Absolute structure parameter: −0.012 (16)

### 

Data collection: *APEX2* (Bruker, 2008 ▶); cell refinement: *SAINT* (Bruker, 2008 ▶); data reduction: *SAINT*; program(s) used to solve structure: *SHELXS97* (Sheldrick, 2008 ▶); program(s) used to refine structure: *SHELXL2014* (Sheldrick, 2008 ▶); molecular graphics: *XP* in *SHELXTL* (Sheldrick, 2008 ▶); software used to prepare material for publication: *XCIF* (Sheldrick, 2008 ▶), *enCIFer* (Allen *et al.*, 2004 ▶) and *publCIF* (Westrip, 2010 ▶).

## Supplementary Material

Crystal structure: contains datablock(s) I, global. DOI: 10.1107/S1600536814024702/hb7309sup1.cif

Structure factors: contains datablock(s) I. DOI: 10.1107/S1600536814024702/hb7309Isup2.hkl

Supporting information file. DOI: 10.1107/S1600536814024702/hb7309Isup3.cml

**Fig. 1.**
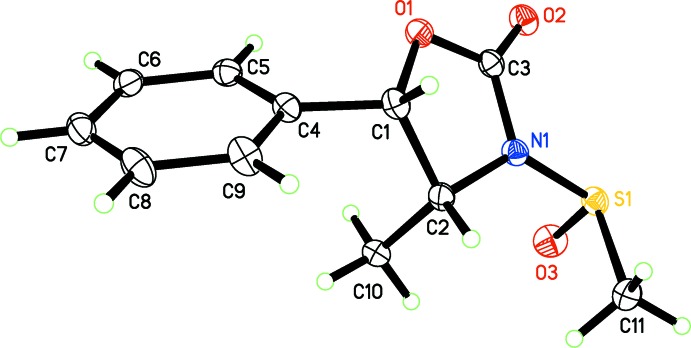
Labelling scheme for (4R,5S)-4-methyl-3-methylsulfinyl-5-phenyl-1,3-oxazolidin-2-one. Thermal displacement ellipsoids are depicted at the 50% probability level.

R S . DOI: 10.1107/S1600536814024702/hb7309fig1.tif
Labelling scheme for (4*R*,5*S*)-4-methyl-3-methylsulfinyl-5-phenyl-1,3-oxazolidin-2-one. Thermal displacement ellipsoids are depicted at the 50% probability level.

CCDC reference: 1033536

Additional supporting information:  crystallographic information; 3D view; checkCIF report

## supplementary crystallographic information

### S1. Introduction

Our research group has inter­est in the development of new methodologies to the synthesis of chiral sulfur compounds (Silveira & Marino, 2013). Thus, we have been preparing chiral oxazolidinones (Silveira, Oliver & Noll, 2013) to the synthesis of asymmetric indole derivatives (Pozza Silveira *et al.*, 2012) as precursors to the preparation of naturally occurring alkaloids (Marino *et al.*, 1992).

### S2. Experimental

Experimental discussion

### S2.1. Synthesis and crystallization

411 mg of (4*R*,5*S*)-4-methyl-5-phenyl­oxazolidin-2-one (2.32 mmol) and 25 mL of dry THF were added to a 50 mL flame-dried round bottom flask charged with argon gas at 0 °C. To this 1.21 mL of *n*-buthyl lithium (1.83 M, 2.21 mmol) was added dropwise into the solution during five minutes and the mixture obtained cooled to -78 °C. Subsequently, 320 mg of sulfinyl chloride was added. After 10 min. the reaction was quenched with 6.5 mL of saturated NH_4_Cl solution. The aqueous layer was extracted with 25 mL of ethyl acetate and the organic phase washed with 8 mL of saturated NaHCO_3_ solution and 10 mL of saturated NaCl solution, respectively. The organic phase was dried over Na_2_SO_4_ and the salt removed by filtration. The solvent was removed under reduced pressure to give a white solid. The crude solid was dissolved with ethyl acetate and hexanes were added dropwise to the solution until a cloudy suspension was observed. The ethyl acetate / hexanes solution was left overnight to evaporate yielding 238 mg of clear colorless rods (45%).

### S2.2. Refinement

Crystal data, data collection and structure refinement details are summarized in Table 1. All non-hydrogen atoms were refined with anisotropic thermal displacement parameters. Hydrogen atoms were included in geometrically calculated positions riding on the carbon to which they are bonded. C—H bond distances were restrained to 0.95 Å (aromatic), 0.98 Å (methyl) and 1.00 Å (methyne). Hydrogen thermal parameters were set as *U*_iso_(H) = 1.2 × *U*_eq_(C)aromatic/methyne and 1.5 × *U*_eq_(C)methyl.

The absolute stereochemistry was determined both by the known chiralty that was retained during synthesis and by comparison of intensities of Friedel pairs of reflections. Both a direct measurement in the differences in intensities (Flack *x* paramter = -0.012 (3), (Parsons *et al.*, 2013)) and a statistical analysis (Hooft *y* parameter = -0.015 (17), Hooft *et al.*, 2008) corroborate that the correct enanti­omorph of the space group and hence handedness of the molecule were determined. All three techniques agree and the correct chirality is shown.

### S3. Results and discussion

The structure of the oxazolidinone is as expected. The steroechemistry from the parent rea­cta­nts was retained through the synthesis. Surprisingly, no significant inter­molecular inter­actions are observed in the crystal. The phenyl group which could exhibit either π···π inter­actions or C—H···π inter­actions shows no sign or indication of such arrangements.

### Crystal data

**Table:**

C_11_H_13_NO_3_S	*D*_x_ = 1.415 Mg m^−^^3^
*M**_r_* = 239.28	Mo *K*α radiation, λ = 0.71073 Å
Orthorhombic, *P*2_1_2_1_2_1_	Cell parameters from 9865 reflections
*a* = 6.1605 (4) Å	θ = 3.2–31.4°
*b* = 11.8490 (8) Å	µ = 0.28 mm^−^^1^
*c* = 15.3861 (11) Å	*T* = 100 K
*V* = 1123.12 (13) Å^3^	Rod, colorless
*Z* = 4	0.22 × 0.09 × 0.06 mm
*F*(000) = 504	

### Data collection

**Table:**

Bruker X8 APEXII CCD diffractometer	3761 independent reflections
Radiation source: fine-focus sealed tube	3507 reflections with *I* > 2σ(*I*)
Graphite monochromator	*R*_int_ = 0.032
Detector resolution: 8.33 pixels mm^-1^	θ_max_ = 31.6°, θ_min_ = 2.2°
φ and ω scans	*h* = −8→9
Absorption correction: multi-scan (*SADABS*; Sheldrick, 2007)	*k* = −17→17
*T*_min_ = 0.707, *T*_max_ = 0.746	*l* = −21→22
30938 measured reflections	

### Refinement

**Table:**

Refinement on *F*^2^	Hydrogen site location: inferred from neighbouring sites
Least-squares matrix: full	H-atom parameters constrained
*R*[*F*^2^ > 2σ(*F*^2^)] = 0.027	*w* = 1/[σ^2^(*F*_o_^2^) + (0.0431*P*)^2^ + 0.127*P*] where *P* = (*F*_o_^2^ + 2*F*_c_^2^)/3
*wR*(*F*^2^) = 0.070	(Δ/σ)_max_ = 0.001
*S* = 1.03	Δρ_max_ = 0.31 e Å^−^^3^
3761 reflections	Δρ_min_ = −0.18 e Å^−^^3^
147 parameters	Absolute structure: Flack *x* determined using 1431 quotients [(*I*^+^)-(*I*^-^)]/[(*I*^+^)+(*I*^-^)] (Parsons *et al.*, 2013)
0 restraints	Absolute structure parameter: −0.012 (16)

### Special details

**Table:**

Geometry. All e.s.d.'s (except the e.s.d. in the dihedral angle between two l.s. planes) are estimated using the full covariance matrix. The cell e.s.d.'s are taken into account individually in the estimation of e.s.d.'s in distances, angles and torsion angles; correlations between e.s.d.'s in cell parameters are only used when they are defined by crystal symmetry. An approximate (isotropic) treatment of cell e.s.d.'s is used for estimating e.s.d.'s involving l.s. planes.

### Fractional atomic coordinates and isotropic or equivalent isotropic displacement parameters (Å^2^)

**Table:**

	*x*	*y*	*z*	*U*_iso_*/*U*_eq_	
S1	0.97856 (6)	0.27012 (3)	0.33636 (2)	0.01721 (9)	
N1	0.8121 (2)	0.16070 (11)	0.31202 (8)	0.0153 (2)	
O1	0.74140 (17)	0.00157 (9)	0.24121 (6)	0.0175 (2)	
O2	1.04668 (18)	0.09415 (9)	0.20595 (7)	0.0194 (2)	
O3	1.13406 (18)	0.23613 (11)	0.40470 (7)	0.0241 (2)	
C1	0.5547 (2)	0.02282 (12)	0.29661 (9)	0.0156 (3)	
H1	0.4376	0.0589	0.2612	0.019*	
C2	0.6387 (2)	0.10893 (12)	0.36433 (9)	0.0152 (3)	
H2	0.5236	0.1659	0.3775	0.018*	
C3	0.8831 (2)	0.08716 (12)	0.24861 (9)	0.0154 (3)	
C4	0.4715 (2)	−0.08568 (12)	0.33334 (9)	0.0155 (2)	
C5	0.5964 (2)	−0.18255 (12)	0.33741 (10)	0.0169 (2)	
H5	0.7380	−0.1831	0.3130	0.020*	
C6	0.5153 (3)	−0.27897 (12)	0.37712 (9)	0.0190 (3)	
H6	0.6021	−0.3451	0.3803	0.023*	
C7	0.3081 (3)	−0.27882 (14)	0.41209 (9)	0.0208 (3)	
H7	0.2528	−0.3447	0.4394	0.025*	
C8	0.1815 (3)	−0.18222 (14)	0.40706 (10)	0.0221 (3)	
H8	0.0387	−0.1823	0.4304	0.026*	
C9	0.2623 (2)	−0.08596 (14)	0.36827 (10)	0.0204 (3)	
H9	0.1755	−0.0198	0.3653	0.025*	
C10	0.7241 (3)	0.05758 (13)	0.44793 (10)	0.0189 (3)	
H10A	0.8343	0.0006	0.4342	0.028*	
H10B	0.6043	0.0221	0.4796	0.028*	
H10C	0.7889	0.1169	0.4840	0.028*	
C11	0.7753 (3)	0.35377 (13)	0.38865 (10)	0.0211 (3)	
H11A	0.7344	0.3184	0.4439	0.032*	
H11B	0.6474	0.3592	0.3510	0.032*	
H11C	0.8327	0.4295	0.3997	0.032*	

### Atomic displacement parameters (Å^2^)

**Table:**

	*U*^11^	*U*^22^	*U*^33^	*U*^12^	*U*^13^	*U*^23^
S1	0.01616 (15)	0.01850 (15)	0.01696 (15)	−0.00282 (12)	−0.00025 (12)	0.00138 (12)
N1	0.0135 (5)	0.0171 (5)	0.0154 (5)	−0.0015 (4)	0.0024 (4)	0.0005 (4)
O1	0.0169 (5)	0.0178 (5)	0.0179 (5)	−0.0019 (4)	0.0058 (4)	−0.0002 (4)
O2	0.0158 (5)	0.0222 (5)	0.0202 (5)	0.0013 (4)	0.0045 (4)	0.0026 (4)
O3	0.0191 (5)	0.0288 (6)	0.0245 (5)	0.0005 (5)	−0.0068 (4)	−0.0011 (5)
C1	0.0114 (6)	0.0180 (6)	0.0174 (6)	0.0006 (5)	0.0021 (5)	0.0025 (5)
C2	0.0136 (6)	0.0147 (6)	0.0172 (6)	0.0011 (5)	0.0045 (5)	0.0010 (5)
C3	0.0147 (6)	0.0162 (6)	0.0152 (6)	0.0017 (5)	−0.0004 (5)	0.0027 (5)
C4	0.0133 (5)	0.0183 (6)	0.0149 (5)	−0.0014 (5)	−0.0004 (5)	0.0014 (5)
C5	0.0160 (6)	0.0185 (6)	0.0160 (6)	−0.0004 (5)	0.0011 (5)	−0.0016 (5)
C6	0.0243 (7)	0.0161 (6)	0.0166 (6)	−0.0003 (6)	−0.0016 (5)	−0.0021 (5)
C7	0.0245 (7)	0.0225 (7)	0.0155 (6)	−0.0078 (6)	−0.0025 (5)	0.0029 (6)
C8	0.0148 (6)	0.0311 (8)	0.0203 (7)	−0.0040 (6)	0.0007 (5)	0.0065 (6)
C9	0.0140 (6)	0.0252 (7)	0.0221 (7)	0.0015 (6)	−0.0004 (5)	0.0060 (6)
C10	0.0229 (7)	0.0185 (7)	0.0153 (6)	−0.0003 (6)	0.0031 (6)	0.0003 (5)
C11	0.0240 (7)	0.0173 (6)	0.0220 (7)	0.0020 (6)	0.0001 (6)	0.0004 (6)

### Geometric parameters (Å, º)

**Table:**

S1—O3	1.4783 (12)	C5—C6	1.389 (2)
S1—N1	1.6948 (13)	C5—H5	0.9500
S1—C11	1.7882 (16)	C6—C7	1.385 (2)
N1—C3	1.3791 (18)	C6—H6	0.9500
N1—C2	1.4716 (18)	C7—C8	1.387 (2)
O1—C3	1.3428 (17)	C7—H7	0.9500
O1—C1	1.4534 (16)	C8—C9	1.380 (2)
O2—C3	1.2056 (17)	C8—H8	0.9500
C1—C4	1.4952 (19)	C9—H9	0.9500
C1—C2	1.547 (2)	C10—H10A	0.9800
C1—H1	1.0000	C10—H10B	0.9800
C2—C10	1.517 (2)	C10—H10C	0.9800
C2—H2	1.0000	C11—H11A	0.9800
C4—C5	1.3832 (19)	C11—H11B	0.9800
C4—C9	1.396 (2)	C11—H11C	0.9800
			
O3—S1—N1	109.93 (7)	C4—C5—H5	119.9
O3—S1—C11	106.55 (7)	C6—C5—H5	119.9
N1—S1—C11	95.73 (7)	C7—C6—C5	120.08 (14)
C3—N1—C2	110.71 (12)	C7—C6—H6	120.0
C3—N1—S1	116.62 (10)	C5—C6—H6	120.0
C2—N1—S1	129.58 (10)	C6—C7—C8	119.83 (14)
C3—O1—C1	109.49 (11)	C6—C7—H7	120.1
O1—C1—C4	110.13 (11)	C8—C7—H7	120.1
O1—C1—C2	104.16 (11)	C9—C8—C7	120.21 (14)
C4—C1—C2	115.28 (11)	C9—C8—H8	119.9
O1—C1—H1	109.0	C7—C8—H8	119.9
C4—C1—H1	109.0	C8—C9—C4	120.09 (14)
C2—C1—H1	109.0	C8—C9—H9	120.0
N1—C2—C10	112.29 (12)	C4—C9—H9	120.0
N1—C2—C1	98.58 (11)	C2—C10—H10A	109.5
C10—C2—C1	114.99 (12)	C2—C10—H10B	109.5
N1—C2—H2	110.2	H10A—C10—H10B	109.5
C10—C2—H2	110.2	C2—C10—H10C	109.5
C1—C2—H2	110.2	H10A—C10—H10C	109.5
O2—C3—O1	123.30 (13)	H10B—C10—H10C	109.5
O2—C3—N1	127.34 (14)	S1—C11—H11A	109.5
O1—C3—N1	109.34 (11)	S1—C11—H11B	109.5
C5—C4—C9	119.60 (13)	H11A—C11—H11B	109.5
C5—C4—C1	122.67 (12)	S1—C11—H11C	109.5
C9—C4—C1	117.65 (13)	H11A—C11—H11C	109.5
C4—C5—C6	120.18 (13)	H11B—C11—H11C	109.5
			
O3—S1—N1—C3	−88.16 (11)	C2—N1—C3—O2	−165.15 (14)
C11—S1—N1—C3	161.92 (11)	S1—N1—C3—O2	−3.1 (2)
O3—S1—N1—C2	69.87 (14)	C2—N1—C3—O1	13.73 (15)
C11—S1—N1—C2	−40.05 (14)	S1—N1—C3—O1	175.77 (9)
C3—O1—C1—C4	−145.05 (12)	O1—C1—C4—C5	19.91 (18)
C3—O1—C1—C2	−20.89 (14)	C2—C1—C4—C5	−97.55 (16)
C3—N1—C2—C10	96.80 (14)	O1—C1—C4—C9	−163.13 (12)
S1—N1—C2—C10	−62.25 (16)	C2—C1—C4—C9	79.41 (16)
C3—N1—C2—C1	−24.78 (14)	C9—C4—C5—C6	−0.9 (2)
S1—N1—C2—C1	176.17 (11)	C1—C4—C5—C6	175.97 (13)
O1—C1—C2—N1	26.31 (13)	C4—C5—C6—C7	0.6 (2)
C4—C1—C2—N1	147.08 (12)	C5—C6—C7—C8	0.2 (2)
O1—C1—C2—C10	−93.27 (14)	C6—C7—C8—C9	−0.8 (2)
C4—C1—C2—C10	27.50 (17)	C7—C8—C9—C4	0.5 (2)
C1—O1—C3—O2	−175.68 (13)	C5—C4—C9—C8	0.4 (2)
C1—O1—C3—N1	5.38 (15)	C1—C4—C9—C8	−176.66 (14)

## References

[bb1] Allen, F. H., Johnson, O., Shields, G. P., Smith, B. R. & Towler, M. (2004). *J. Appl. Cryst.* **37**, 335–338.

[bb2] Bruker (2008). *APEX2* and *SAINT*. Bruker–Nonius AXS Inc., Madison, Wisconsin, USA.

[bb3] Clara-Sosa, A., Pérez, L., Sánchez, M., Melgar-Fernández, R., Juaristi, E., Quintero, L. & Anaya de Parrodi, C. (2004). *Tetrahedron*, **60**, 12147–12152.

[bb4] Evans, D. A., Faul, M. M., Colombo, L., Bisaha, J. J., Clardy, J. & Cherry, D. (1992). *J. Am. Chem. Soc.* **114**, 5977–5985.

[bb5] Hooft, R. W. W., Straver, L. H. & Spek, A. L. (2008). *J. Appl. Cryst.* **41**, 96–103. 10.1107/S0021889807059870 PMC2467520 19461838

[bb6] Marino, J. P., Bogdan, S. & Kimura, K. (1992). *J. Am. Chem. Soc.* **114**, 5566–5572.

[bb7] Parsons, S., Flack, H. D. & Wagner, T. (2013). *Acta Cryst.* B**69**, 249–259. 10.1107/S2052519213010014 PMC3661305 23719469

[bb10] Romanenko, V. D., Thoumazet, C., Lavallo, V., Tham, F. S. & Bertrand, G. (2003). *Chem. Commun.* pp. 1680–1681.

[bb11] Sheldrick, G. M. (2007). *SADABS*. University of Göttingen, Germany.

[bb12] Sheldrick, G. M. (2008). *Acta Cryst.* A**64**, 112–122. 10.1107/S0108767307043930 18156677

[bb13] Silveira, G. P. & Marino, J. P. (2013). *J. Org. Chem.* **78**, 3379–3383. 10.1021/jo302798j 23480349

[bb8] Pozza Silveira, G., Bonfante de Carvallho, C. & Oliver, A. (2012). *Acta Cryst.* E**68**, o2048. 10.1107/S160053681202569X PMC3393316 22807873

[bb9] Pozza Silveira, G., Oliver, A. G. & Noll, B. C. (2013). *Acta Cryst.* E**69**, o979. 10.1107/S1600536813014001 PMC3685113 23795132

[bb14] Westrip, S. P. (2010). *J. Appl. Cryst.* **43**, 920–925.

