# Supplementary figures and images for: Crystal structure of (4R,5S)-4-methyl-3-methyl­sulfinyl-5-phenyl-1,3-oxazolidin-2-one
Source: Acta Crystallogr Sect E Struct Rep Online. 2014 Nov 15;70(Pt 12):o1257–8. doi: 10.1107/S1600536814024702 (PMC4257383; doi:10.1107/S1600536814024702)

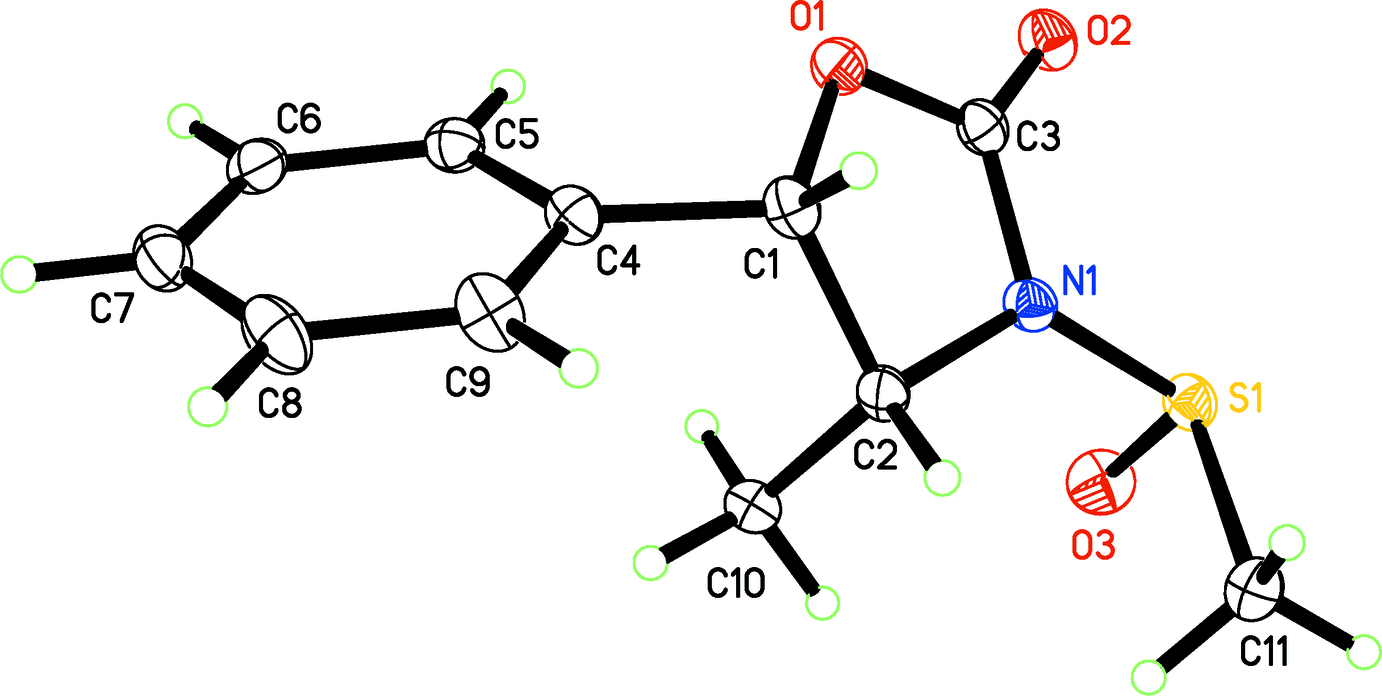

Supplement: Supplementary file 4 [file e-70-o1257-fig1.tif]
